# Supplementary material for: Autonomic nervous system responses of dogs to human-dog interaction videos
Source: PLoS One. 2022 Nov 3;17(11):e0257788. doi: 10.1371/journal.pone.0257788 (PMC9632911; doi:10.1371/journal.pone.0257788)
Supplement: S1 Fig — An electrode gel was added to the electrode. The negative electrode was placed slightly below the sternal peduncle. The grounding electrode is separated from the positive electrode. The positive electrode was attached around the processus xiphoideus. (DOCX) [file pone.0257788.s001.docx]

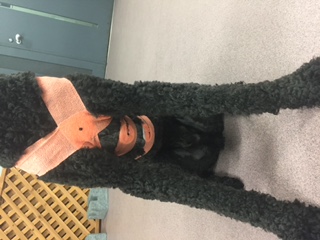

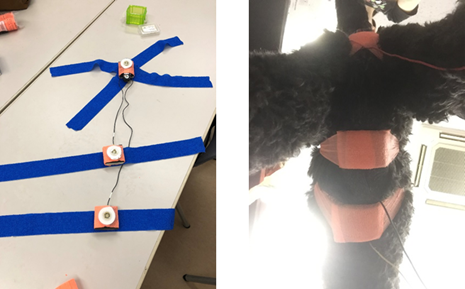


**S1 Fig. Heart rate measurement procedure.** An electrode gel was added to the electrode. The negative electrode was placed slightly below the sternal peduncle. The grounding electrode is separated from the positive electrode. The positive electrode was attached around the processus xiphoideus.
