# Supplementary figures and images for: Autonomic nervous system responses of dogs to human-dog interaction videos
Source: PLoS One. 2022 Nov 3;17(11):e0257788. doi: 10.1371/journal.pone.0257788 (PMC9632911; doi:10.1371/journal.pone.0257788)

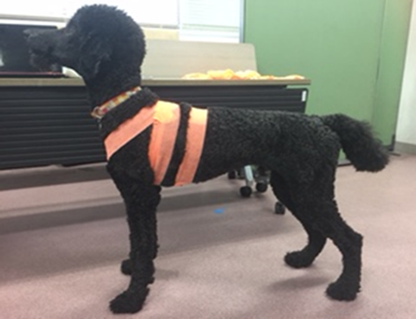


**S2 Fig. After the electrocardiograph is placed on the dog.**

Supplement: S2 Fig — (DOCX) [file pone.0257788.s002.docx]
