# Supplementary material for: Autonomic nervous system responses of dogs to human-dog interaction videos
Source: PLoS One. 2022 Nov 3;17(11):e0257788. doi: 10.1371/journal.pone.0257788 (PMC9632911; doi:10.1371/journal.pone.0257788)
Supplement: S1 Table — (DOCX) [file pone.0257788.s003.docx]

**S1 Table. Statistics of the results of the LMMs between resting conditions.**

| HRV | fixed effect | Estimate | Std. Error | *df* | *t* value | *p* value |
| --- | --- | --- | --- | --- | --- | --- |
| meanRRI | day | 0.02 | 0.05 | 27.62 | 0.35 | 0.729 |
|  | condition | -0.01 | 0.02 | 33.75 | -0.21 | 0.838 |
|  | interaction | 0.02 | 0.05 | 27.62 | 0.44 | 0.665 |
| RMSSD | day | -0.04 | 0.19 | 22.00 | -0.19 | 0.852 |
|  | condition | -0.04 | 0.09 | 32.89 | -0.44 | 0.662 |
|  | interaction | 0.20 | 0.17 | 22.00 | 1.17 | 0.254 |
| SDNN | day | -0.16 | 0.11 | 22.00 | -1.54 | 0.138 |
|  | condition | 0.04 | 0.06 | 30.40 | 0.64 | 0.527 |
|  | interaction | 0.11 | 0.10 | 22.00 | 1.11 | 0.280 |
