# Supplementary material for: Autonomic nervous system responses of dogs to human-dog interaction videos
Source: PLoS One. 2022 Nov 3;17(11):e0257788. doi: 10.1371/journal.pone.0257788 (PMC9632911; doi:10.1371/journal.pone.0257788)
Supplement: S2 Table — (DOCX) [file pone.0257788.s004.docx]

**S2 Table.** **Statistics of the results of the LMMs between event conditions.**

| HRV | fixed effect | Estimate | Std. Error | *df* | *t* value | *p* value |
| --- | --- | --- | --- | --- | --- | --- |
| meanRRI | day | 0.05 | 0.03 | 19.32 | 2.01 | 0.058 |
|  | condition | -0.04 | 0.01 | 10.92 | -3.16 | 0.009 |
|  | interaction | 0.01 | 0.04 | 33.42 | 0.39 | 0.703 |
| RMSSD | day | -0.01 | 0.07 | 21.38 | -0.14 | 0.894 |
|  | condition | -0.11 | 0.04 | 11.14 | -2.80 | 0.017 |
|  | interaction | 0.07 | 0.11 | 30.12 | 0.66 | 0.517 |
| SDNN | day | -0.01 | 0.06 | 31.09 | -0.26 | 0.798 |
|  | condition | -0.07 | 0.03 | 15.95 | -2.44 | 0.027 |
|  | interaction | 0.09 | 0.08 | 42.75 | 1.11 | 0.275 |
