# Supplementary material for: Autonomic nervous system responses of dogs to human-dog interaction videos
Source: PLoS One. 2022 Nov 3;17(11):e0257788. doi: 10.1371/journal.pone.0257788 (PMC9632911; doi:10.1371/journal.pone.0257788)
Supplement: S3 Table — (DOCX) [file pone.0257788.s005.docx]

**S3 Table.** **Statistics of multiple comparisons between event conditions by paired t-test.**

| HRV | condition | *df* | *t* value | adjusted *p* value | effect size(*r*) | 95% Cl |
| --- | --- | --- | --- | --- | --- | --- |
| meanRRI | OW-A-IGN vs OW-A-INT | 11 | -0.86 | 0.814 | 0.25 | -0.10 to 0.05 |
|  | OW-A-IGN vs NOW-A-INT | 11 | -1.85 | 0.459 | 0.49 | -0.12 to 0.01 |
|  | OW-A-IGN vs OW-S-INT | 11 | -2.99 | 0.073 | 0.67 | -0.16 to -0.02 |
|  | OW-A-INT vs NOW-A-INT | 11 | -0.56 | 0.588 | 0.17 | -0.12 to 0.07 |
|  | OW-A-INT vs OW-S-INT | 11 | -1.33 | 0.632 | 0.37 | -0.17 to 0.04 |
|  | NOW-A-INT vs OW-S-INT | 11 | -1.60 | 0.550 | 0.43 | -0.09 to 0.01 |
| RMSSD | OW-A-IGN vs OW-A-INT | 11 | -1.59 | 0.564 | 0.43 | -0.39 to 0.06 |
|  | OW-A-IGN vs NOW-A-INT | 11 | -3.14 | 0.047 | 0.69 | -0.40 to -0.07 |
|  | OW-A-IGN vs OW-S-INT | 11 | -3.23 | 0.048 | 0.70 | -0.56 to -0.10 |
|  | OW-A-INT vs NOW-A-INT | 11 | -0.61 | 0.552 | 0.18 | -0.33 to 0.12 |
|  | OW-A-INT vs OW-S-INT | 11 | -1.32 | 0.425 | 0.37 | -0.45 to 0.11 |
|  | NOW-A-INT vs OW-S-INT | 11 | -1.33 | 0.629 | 0.37 | -0.26 to 0.06 |
| SDNN | OW-A-IGN vs OW-A-INT | 11 | -1.99 | 0.287 | 0.51 | -0.34 to 0.02 |
|  | OW-A-IGN vs NOW-A-INT | 11 | -3.18 | 0.044 | 0.69 | -0.31 to -0.06 |
|  | OW-A-IGN vs OW-S-INT | 11 | -3.34 | 0.040 | 0.71 | -0.38 to -0.08 |
|  | OW-A-INT vs NOW-A-INT | 11 | -0.24 | 0.818 | 0.07 | -0.24 to 0.20 |
|  | OW-A-INT vs OW-S-INT | 11 | -0.74 | 1.422 | 0.22 | -0.27 to 0.14 |
|  | NOW-A-INT vs OW-S-INT | 11 | -0.73 | 0.962 | 0.21 | -0.18 to 0.09 |
