# Supplementary material for: Autonomic nervous system responses of dogs to human-dog interaction videos
Source: PLoS One. 2022 Nov 3;17(11):e0257788. doi: 10.1371/journal.pone.0257788 (PMC9632911; doi:10.1371/journal.pone.0257788)
Supplement: S4 Table — The explanatory variables are variables selected by the stepwise method. (DOCX) [file pone.0257788.s006.docx]

**S4 Table.** **Parameter estimates for the relationship using HRV (OW-S-INT data minus the OW-A-INT data) data as the dependent variable and C-BARQ scores as the explanatory variable.**

| HRV | Explanatory variables | Estimate | Std. Error | *t* value | *p* value |
| --- | --- | --- | --- | --- | --- |
| meanRRI | attachment or attention-seeking behavior | -0.62 | 0.26 | -2.41 | 0.04 |
|  | dog-directed aggression | -0.34 | 0.26 | -1.29 | 0.23 |
|  | dog-directed fear | 0.52 | 0.27 | 1.91 | 0.09 |
|  | R squared value | 0.35 |  |  |  |
| RMSSD | attachment or attention-seeking behavior | -0.35 | 0.30 | -1.18 | 0.27 |
|  | dog-directed aggression | -0.46 | 0.31 | -1.50 | 0.17 |
|  | dog-directed fear | 0.43 | 0.32 | 1.36 | 0.21 |
|  | R squared value | 0.11 |  |  |  |
| SDNN | attachment or attention-seeking behavior | -0.10 | 0.33 | -0.31 | 0.77 |
|  | dog-directed aggression | -0/19 | 0.34 | -0.543 | 0.60 |
|  | dog-directed fear | 0.50 | 0.35 | 1.42 | 0.19 |
|  | R squared value | 0.20 |  |  |  |
