# Supplementary material for: Autonomic nervous system responses of dogs to human-dog interaction videos
Source: PLoS One. 2022 Nov 3;17(11):e0257788. doi: 10.1371/journal.pone.0257788 (PMC9632911; doi:10.1371/journal.pone.0257788)
Supplement: Attachment — Submitted filename: Response to Reviewers.docx [file pone.0257788.s009.docx]

We appreciate the time and effort you and each of the reviewers have dedicated to providing insightful feedback on how to enhance our paper. We have noted below the points you have raised.

Reviewer #1: Dear authors,

thanks for addressing my comments and those of the other reviewers (and the editor).

I am satisfied with the replies and modifications you provided. One suggestion, it would be easier if next time you'll indicate the lines where you modified your manuscript in our response.

Re: Thank you for your comment. The lines of the manuscript modified are listed below.

The modified lines in Abstract are 42-46,48.42-46,48.

The modified lines in Introduction are 79-86,95,96,98,113,134,135,145,146,163-177.

The modified lines in Method are 186-188,234,235,248-252,311-315,317,318,352-358.

The modified lines in Results are 379-386,388,389 and Table2, Table3, S3 Table, S4 Table.

The modified lines in Discussion are 411-413,419,420,422,425-435,439-444,450-453,455,456,468,470-472,491-496.

Also, the references added are 4-10,39,51. The references have been renumbered accordingly.
