# Supplementary material for: Autonomic nervous system responses of dogs to human-dog interaction videos
Source: PLoS One. 2022 Nov 3;17(11):e0257788. doi: 10.1371/journal.pone.0257788 (PMC9632911; doi:10.1371/journal.pone.0257788)
Supplement: Attachment — Submitted filename: Response to Reviewers_1016.docx [file pone.0257788.s010.docx]

We appreciate the time and effort you and each of the reviewers have dedicated to providing insightful feedback on how to enhance our paper. We have reviewed the reference lists and ensured that it is complete and correct. The references added are 4-9,38,49, and the reference deleted is the previous number 32 because of changes in the text. The references have been renumbered accordingly. We also added the normality test statistic to line 327 and the effect sizes and Cl to lines 355-359 (also S3Table) of the revised manuscript. Other points raised are noted below.

From Editor and Editorial office.

Please review your reference list to ensure that it is complete and correct. If you have cited papers that have been retracted, please include the rationale for doing so in the manuscript text, or remove these references and replace them with relevant current references. Any changes to the reference list should be mentioned in the rebuttal letter that accompanies your revised manuscript. If you need to cite a retracted article, indicate the article’s retracted status in the References list and also include a citation and full reference for the retraction notice.

Re: Thank you for your comment. As pointed out by the reviewer, added a reference on self-recognition in other animals (References list: 4-9) to the Introduction. Also, as pointed out by the reviewer, we added evidence on the relationship between temperament and social behavior in dogs (References list: 38) and changed the text accordingly, so we deleted reference list-32 from the Introduction. As well, the reviewer pointed out, we added a reference that the dog showed increased heart rate, and decreased parasympathetic activity during the reunion with the owner after a short time of separation (References list: 49) to the Discussion.

I’d highly suggest the authors to report all stats parameters (CIs, effect sizes, assumptions check etc.)

Re: Thank you for your comment. We included the statistical parameters as you suggested, and we revised the tables.

From Reviewer #1:

Thanks for addressing my comments and those of the other reviewers (and the editor).

I am satisfied with the replies and modifications you provided. One suggestion, it would be easier if next time you'll indicate the lines where you modified your manuscript in our response.

Re: Thank you for your comment. The lines of the manuscript modified are listed below.

The modified lines in Abstract are 42-46,48.

The modified lines in Introduction are 79-86,95,96,98,113,134,135,145,146,163-177.

The modified lines in Method are 186-188,234,235,248-252,311-315,317,318,352-358,340.

The modified lines in Results are 371,373,379-386,388,389 and Table2, Table3, S3 Table, S4 Table.

The modified lines in Discussion are 411-413,419,420,422,425-435,439-444,450-453,455,456,468,470-472,491-496.
